# Use of Silicon T-Tube for Subglottic Stenosis and Tracheal Stenosis

**DOI:** 10.22038/IJORL.2023.67492.3308

**Published:** 2023-05

**Authors:** Chethana Ramesh, Arun Dehadaray, Maitri Kaushik, Prasun Mishra, Simran Sidhu

**Affiliations:** 1 *Department of ENT, Bharati Vidyapeet (Deemed to be) University Medical College, Pune, Maharashtra, India.*

**Keywords:** Silicon T-tube, Subglottic stenosis, Tracheal stenosis

## Abstract

**Introduction:**

The management of subglottic and tracheal stenosis is challenging for any ENT surgeon. The treatment choice depends on the site, severity of stenosis, patient symptoms, and surgeon preferences. The various options for the management include endoscopic balloon dilatation, various types of laryngotracheoplasty, resection anastomosis, and insertion of a silicon T-tube. Compared to the above, silicon T-tube stenting is a better alternative, as it is a onetime procedure, easy to perform with fewer chances of complications. Shiann Yann lee technique is a form of laryngotracheoplasty with long-term stenting using silicon T-tube. This article analyzed our silicon T-Tube insertion result in patients with subglottic and tracheal stenosis using this technique.

**Materials and Methods:**

In this retrospective study, we included a total of 21 patients with subglottic and tracheal stenosis who underwent silicon T-Tube insertion. Data regarding the site of stenosis, procedure, complications, and outcome were analyzed.

**Results:**

Out of 21 patients, nine patients had subglottic stenosis (42.8%), 8 had cervical tracheal stenosis (38.09%), 3 had thoracic tracheal stenosis (14.28%), and 1 (4.7%) had combined subglottic and cervical tracheal stenosis. Out of 21 patients,7 (33.3%) have undergone successful removal of silicon T-Tube so far, one death due to medical reasons, and 13 patients (61.9%) are still on Silicon tube on regular follow-up. They are comfortable with the tube in situ.

**Conclusions:**

Silicon T-Tube for benign acquired laryngotracheal stenosis with Shiann Yann Lee's technique is effective, safe with less complication, and good acceptability and tolerance by the patient.

## Introduction

Laryngotracheal stenosis can be a life-threatening problem (1). The upper airway's anatomical structure comprises the larynx, including supraglottis, glottis, subglottis, and trachea. Subglottic/ tracheal stenosis can occur due to trauma resulting from endotracheal intubation, burns, infectious processes, and autoimmune diseases. This subglottic and tracheal stenosis may be asymptomatic, or it can lead to life-threatening consequences like respiratory distress/failure, cardiopulmonary arrest, and even death. The management of subglottic stenosis and tracheal stenosis remains a challenge. The various options for the management include endoscopic balloon dilatation, laryngotracheoplasty, resection anastomosis, and insertion of silicon T-tube (2). Treatment choice depends on the severity of symptoms (stridor), site, stenosis size, and surgeon preferences. The patient's socioeconomic status also plays a significant role in selecting treatment options. Endoscopic balloon dilatation though expensive, does not preclude the requirement of open surgical intervention and tracheostomy dependence. The procedure may require multiple settings as well. Various open surgical techniques like resection anastomosis require a well-prepared airway with good physiological conditions, complex surgical techniques, a need for ICU stay, and have an increased rate of life-threatening complications. In comparison to the above two treatment options, the third one that is silicon T-tube stenting is an alternative that is safe and easy to perform. The procedure is a one-time procedure and has lesser chances of life-threatening complications and patient is non-dependent on tracheostomy tube and has normal speech.

In 1964 William W. Montgomery first described the successful placement of a stent in the airway; however, it was rigid and challenging to manufacture, so one year later, he came up with the flexible, less tissue reaction-inducing silicon T-tube (3,4). These silicon T tubes are made up of medical silicon, which is soft, usually less stimulating to the airway, less tissue reaction like granulation formation, provides good stability without much chance of migration in the airway. It also has good patient compatibility in terms of phonation compared to tracheostomy (5). 

Stents promote airway patency from scar tissue contracture, encourage the development of new epithelial cover, and prevent mechanical injury caused by swallowing and breathing during wound healing (6,7). The Shiann Yann lee technique 8,a form of laryngotracheoplasty with long-term stenting first published in 1993, has revolutionized the management of subglottic and tracheal stenosis. Though the technique was described over three decades back, other than a few minor modifications discussed and published, no significant changes in the technique have been reported so far.

This study aims to assess the treatment outcome of patients with subglottic and tracheal stenosis treated with silicon T-Tube insertion.

## Materials and Methods

This was a retrospective study conducted in a tertiary medical centre in the Western part of India. The study was conducted in 2022, and data over 13 years, from 2009-2022, were collected retrospectively. In this study, we included a total of 21 patients with subglottic and tracheal stenosis who underwent silicon T-Tube insertion. The Patients who underwent other modalities like balloon dilatation or refused other treatment modalities were excluded from the study. All data on demographic characteristics, symptoms, diagnosis, treatment, and outcome status was collected retrospectively through clinical records. The availability and color of the ration card were used as a proxy indicator for socioeconomic status. Yellow cards for those below the poverty line, white for those above the poverty line, and orange for those who fall just above the poverty line were noted. 

Patients presenting with stridor underwent emergency tracheostomy except for 2 cases of thoracic tracheal stenosis that underwent primary silicon T-tube insertion.

**Fig1:**
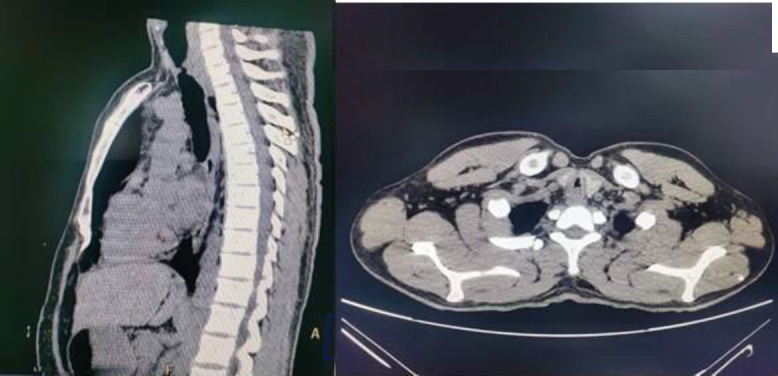
CT scan images showing tracheal stenosis

CT scan neck and/or thorax was done for all the patients. Fig 1 shows a CT scan image of tracheal stenosis. The level of stenosis was classified as subglottis, cervical tracheal, and thoracic tracheal stenosis. Once the diagnosis was confirmed, the patient was posted for silicon T-tube insertion under General anaesthesia.

**Fig 2:**
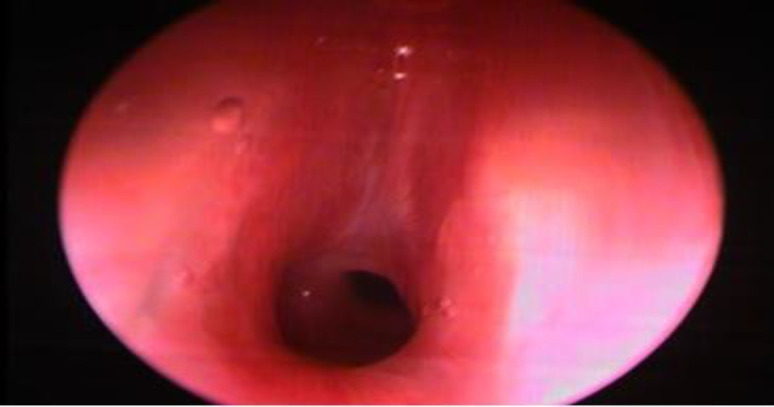
DL scope +0 degree endoscope aided image of subglottic stenosis

General anaesthesia through a tracheostomy tube was given. Once under general anaesthesia, Direct laryngoscopy with 0-degree rigid endoscopy was done to assess the level, length, and severity of stenosis (Fig 2). 

**Fig3:**
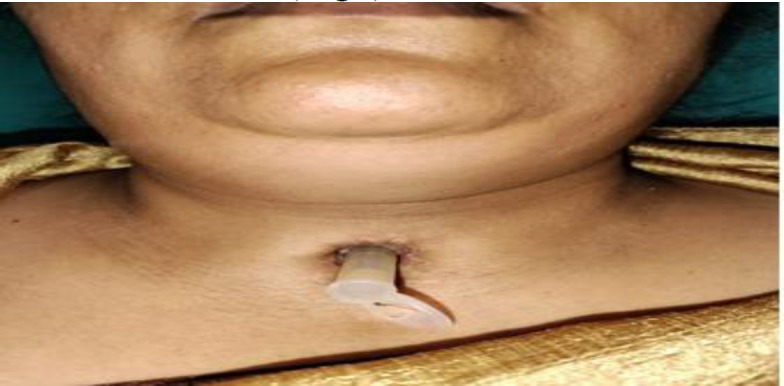
Post op patient of T-tube insertion

For tracheal stenosis, Shiann Yann Lee's technique was followed (8). Midline vertical incision was taken depending upon the level of the stenosis, usually extending from the upper border of the stoma of the tracheostomy site to the lower border of the thyroid cartilage. Once the anterior tracheal wall was exposed, the trachea with underlying stenotic segment was incised. Granulation tissue in the lumen, if any, was removed, fibrous tissue in the lumen was released, and sutures were taken from the incised tracheal wall to the adjacent soft tissue and subcutaneous tissue. In the original procedure described by Shiann Yann Lee, only the mucosa of the trachea is sutured to extraluminal tissues, however with significantly less mucosa available due to stenosis and also due to weak grip to suture the mucosa, we have sutured tracheal wall to extraluminal soft tissues tissue. The appropriate size silicon T tube was chosen and introduced after the removal of the tracheostomy tube such that both the ends of the tube bypasses the stenotic segment by at least 0.3cm, also ensuring that the proximal end is below the level of the glottis and distal end is at least 1cm above the carina. The position of the T –tube was rechecked by introducing a DL scope and 0-degree endoscope after the removal of the extension of the neck. After the insertion of the T-tube, ventilation was continued through the T-tube. To prevent the anaesthetic gas leak from the proximal limb of the T-tube, the laryngeal inlet was temporarily occluded with ribbon gauze. Minimal air leak through proximal end of T-tube was noted despite this. The skin was sutured with Ethilon 3-0. Once the patient was reversed from general anesthesia and the transglottic air flow was tolerated by the patient, the horizontal extraluminal stem of the T-tube was capped so that the patient breathed transnasally on the table itself, and good voice was confirmed. For subglottic stenosis, a similar procedure was followed; however, an anterior cricoid split was done, a part of the cricoid cartilage was removed, and the cut ends were sutured to subcutaneous tissue as before. In upper cervical stenosis, an anterior cricoid split was done without the removal of cartilage. For thoracic tracheal stenosis, dilatation of the soft stenotic segment was done using a bougie, and for the fibrotic stenotic segment, radial cuts were given with cold steel instruments (micro-laryngeal instruments), and then the silicon T-tube was inserted. The patients were kept in the ward for 72 hrs, to watch for respiratory distress and subcutaneous emphysema. Post-op day one, a video laryngoscopy was done in adult patients, and the position of the T-tube was confirmed to be below the level of the glottis. If the position was slightly above expected, then the horizontal limb of the T-tube was pulled down, and a stay suture was placed above it to prevent it from superior migration. 1-2cc of saline was passed through the horizontal stem of the silicon T-tube, and the patient was asked to cough; this procedure was also taught to relatives. This was done for irrigation of the tube and to keep it patent. The subsequent follow-up was after ten days to check for patency of the tube; after instilling 1-2cc of saline, suctioning of the horizontal T-tube, both above and below the horizontal stem was done to ensure patency of the tube. Patients were followed up for every 3 months after that (Fig3). 

Assessment for removal of the T-tube was done after one year of the procedure. An x-ray neck lateral view was performed and space between the trachea and T-tube was assessed; if radiologically there was adequate space, then it was considered favorable for removal. Check Direct laryngoscopy with a 0-degree endoscope was performed to visualize the laryngotracheal tree. Once the resolution of the stenosis was confirmed, saline was applied and the silicon t-tube was removed. A tracheostomy tube was placed for 24 hrs and the patient was decannulated in the ward. This was done to doubly ensure complete resolution of airway edema post operatively. 

However, in subglottic stenosis, assessment for removal of silicon T-tube was started after two years. As it is observed that as the child grows, the subglottis diameter increases, and the child becomes more comfortable and decannulation easier.

## Results

**Fig 4:**
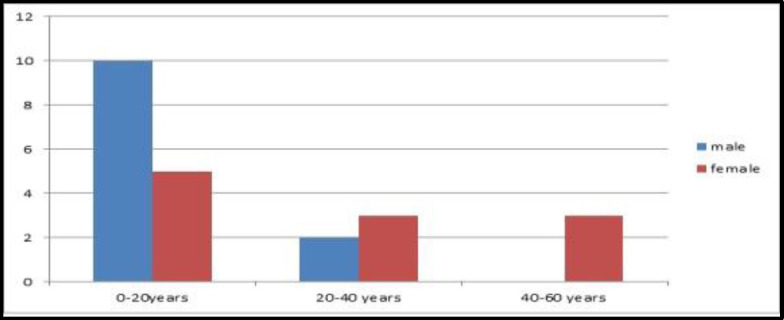
Age and sex distribution.n-21

The minimum age of the patient who underwent the procedure was two years, and the maximum age was 56 years. The mean age was 16.6 years, with a standard deviation-15.83. Age and sex distribution have been given in (Fig4). 

**Fig 5:**
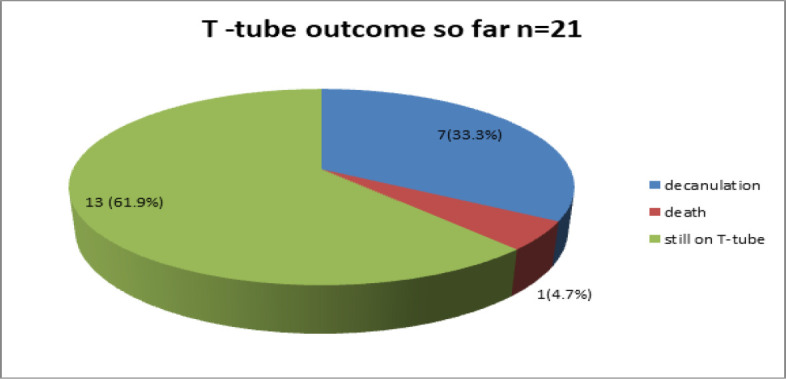
Our outcomes so far

All patients belonged to low socioeconomic strata as indicated by their yellow ration cards. All patients who presented to us had a history of prolonged intubation, of which two were due to COVID. Stridor was the presenting symptom in 16 out of 21 patients (76.10%), whereas 5 out of 21 (23.08%) presented as failure to decannulate/failure to extubate. Out of 21 patients, nine patients had subglottic stenosis (42.8%), 8 had cervical tracheal stenosis (38.09%), 3 had thoracic tracheal stenosis (14.28%), and 1 (4.7%) had combined subglottic and cervical tracheal stenosis. All the subglottic patients had grade 3 or 4 stenosis whereas cervical or thoracic tracheal stenosis had very severe stenosis. Out of 21 patients, one child with subglottic stenosis with silicon t-tube in situ passed away due to other medical reasons one year after the procedure. Seven out of 21 patients (33.3%) have undergone successful removal of silicon T-Tube so far (Fig5). 

Of these, 4 are patients of subglottic stenosis, and 3 are patients of tracheal stenosis (1 thoracic and two cervical tracheal stenoses each). Thirteen patients (61.9%) are still on Silicon tubes, as four were done recently in the last two years and are on regular follow-up, and nine are unwilling for the further procedure due to cost issues for the procedure as most of them are from very low socioeconomic strata and are comfortable to carry out their day to day activities with the tube in situ. One patient with thoracic tracheal stenosis underwent removal eight years after the procedure; however, she developed respiratory distress within 24 hrs, and the silicon T-Tube had to be reintroduced. One child with subglottic stenosis developed granulations at the upper end of the T-tube, the tube was removed, and a tracheostomy tube was introduced. The granulations were removed with a laryngeal microdebrider, and the patient decannulated after keeping the tracheostomy tube in situ for one week. 

**Fig 6:**
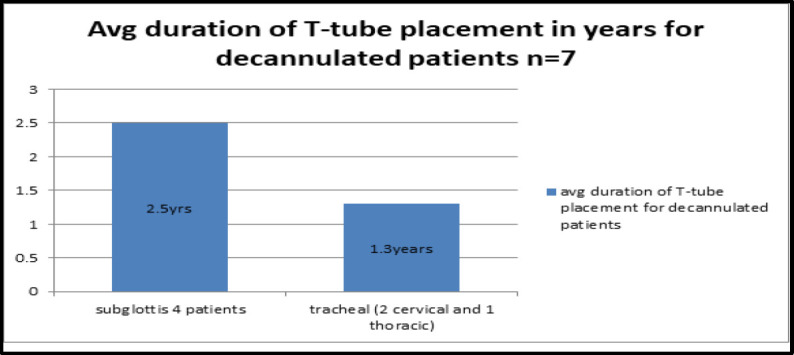
Average duration of T-tube for decannulated patients

Out of all decannulated patients the mean duration until decannulation was 2.5 years for subglottic stenosis patients who underwent decannulation and 1.3 years for tracheal stenosis patients (Fig 6). 

The mean duration of T-tube placement for all the patients decannulated so far is 1.9 years (standard deviation 0.85.)

**Fig 7:**
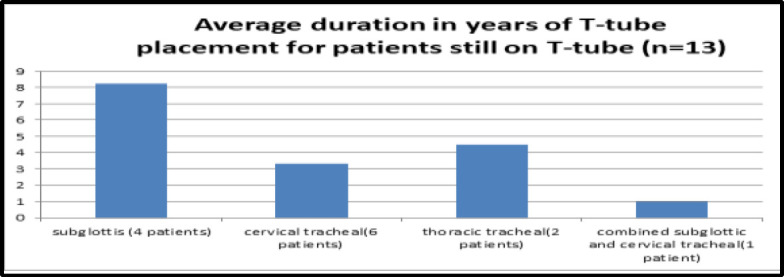
Average duration in years of T-tube placement for patients still on T-tube

As shown in figure 7, the mean duration of the T-tube in place for patients with subglottic stenosis who are not decannulated is eight years, whereas, for cervical and thoracic trachea, it is two years and 4.5 years each. 

The mean duration of T-tube placement for all the patients not decannulated so far is 4.26 years (standard deviation 3.03).

The results are shown in Table 1. The table shows the age, stenosis level, T-tube placement duration, and outcomes.

**Table 1:** Details of individual patients who underwent Silicon T-tube insertion and their outcome

**Pt no**	**Age at presentation**	**sex**	**Year of T-tube insertion**	**Level of stenosis**	**Duration of T-tube placement**	**Outcome**
1	3	male	2013	subglottis	3 months	Decannulated
2	5	female	2018	subglottis	2 years	Decannulated
3	3	male	2016	subglottis	4 years 10 months	Decannulated
4	5	female	2019	subglottis	3 years	Decannulated
5	12	Male	2019	Cervical trachea	1 year 10 months	Decannulated
6	17	male	2019	Cervical trachea	1 year 8 months	Decannulated
7	23	male	2021	Thoracic trachea	6 months	Decannulated
8	3	male	2013	subglottis	1 year	Death due to medical cause
9	9	male	2018	subglottis	3 years 8 months	On T-tube
10	9	male	2013	subglottis	9 years 4 months	On T-tube
11	2	female	2017	Cervical trachea	5 years	On T-tube
12	5	Female	2015	Subglottis	7 years	On T-tube
13	12	male	2016	Cervical trachea	6 years	On T-tube
14	26	Female	2019	Cervical trachea	3 years	On T-tube
15	20	female	2014	Thoracic trachea	8 years	Attempted removal failed, had to reintroduce
16	19	male	2021	Subglottis +cervical trachea	1 year	On T-tube
17	46	female	2022	Cervical trachea	6months	On T-tube
18	25	male	2021	Thoracic trachea	1 year	On T-tube
19	46	female	2022	Cervical trachea	6 months	On T-tube
20	3	male	2009	subglottis	13 years	On T-tube
21	56	female	2017	Cervical trachea	5 years	On T-tube

## Discussion

In the modern era with better ICU setup and a better understanding of endotracheal tube and tracheostomy tube pressure, the rates of tracheal stenosis have drastically reduced. The cuff pressure should be maintained at 20-30mm H2O if > 30mm H20, there is resulting venous stasis and tissue necrosis, with a resulting inflammatory response that leads to stenosis (9). Laryngotracheal stenosis is a challenge to manage for any ENT surgeon. With the advancement of the ventilator, and endotracheal tube management, especially in the neonatal ICU, the subglottic stenosis risk has reduced to approximately 1% (10).

Repeated dilatation using balloon dilators may or may not work, and out-of-pocket expenditure was primary concern to all our patients who are primarily from lower socioeconomic strata. Conservative procedures which result in less tissue damage are better options as they are more physiological. Shiann Yann Lee’s Technique is one such technique. Shiann Yann lee technique is a relatively easy and cost-effective procedure for laryngotracheal stenosis. Meticulously performed surgery with a selection of appropriate size silicon T-tube gives excellent results with rather better acceptance by patients.

Further advantages of silicon T-tube insertion are, it is socially well accepted with maintenance of speech, and nasal respiration. Daily care of the tube is also simple and easily done at home, also less post-operative period in comparison to resection anastomosis or laryngotracheoplasty. The most common complication after T tube surgery was restenosis and granulation at T-tube tip according to Tsai SC et al (11). According to Kurrus JA et al common complications include stent migration (12), blockade of tube because of secretions or inflammatory granulation formation. We had one case of granuloma formation at the T-tube superior end; similarly, the reason for one patient who had to be restented with a t-tube when decannulation was tried after eight years is due to restenosis as a result of ongoing stenosis process in the trachea.

There has been well-documented evidence of tolerance and efficacy of silicon T-tube; however, the duration the stent should be kept in situ is debated. According to Dumon (13), 6-12 months placement for stent was recommended; however, he later recommended keeping it longer to reduce chances of restenosis. Josef-Martinez-Ballaris reported temporary stenting for 18 months (14). According to Kelkar success rate of 89% was reported for stents kept for 8-10 months (15). Decannulation and closure of tracheostoma are one of the primary goals of modern-day therapy addressing airway stenosis (16). In our series, we recommend that a child with subglottic stenosis consider decannulation after two years; as the child grows, subglottis diameter increases, hence easier decannulation. For cervical tracheal stenosis, decannulation is to be considered after one year, whereas for thoracic tracheal stenosis, we recommend keeping the stent for longer or, in certain cases, lifelong (though our experience is limited in this) as it is the most challenging. The mean duration of patients with T-tube who are not decannulated in cases of subglottic stenosis in our series is eight years due to above-mentioned reasons. Such long-term use of silicon T-tube without complications is not discussed widely in the literature. However, in a study by Zaima et al six patients who underwent T-tube insertion as an additional operation after other failed procedures (17), T-tube stenting was kept for an average duration of 65.8months. In our study, though only 33.3 % have undergone successful decannulation so far, the remaining 13(61.9%) patients who are still on T-tube were either performed recently or had refused further evaluation and procedures due to cost issues and due to their comfort to carry out day to day activities or speech with the tube in situ. In a resource-constrained setting success rate of this procedure is measured in terms of average years of being symptom-free. In our study, though 61.9% are still on T-tube for an average of 4.26 years, they are symptom-free and comfortable.

In our study, we cannot confirm whether placing T-tube in situ for a longer period has any significant advantage in terms of outcomes; however, in literature there is no consensus regarding the time duration of T-tube placement (18).

 The data of the 21 patients in our series confirm the excellent tolerance of silicon T-Tube in the airway. There was no other lesion attributable to the stent other than a granuloma noted at the upper end of the tube in one patient. No life-threatening complications arose because of the procedure or the tube. The tube can be kept for a long duration as well as shown in our experience, with no complications or patient-related discomfort. Yet the study has some limitations. The study could not compare silicon T-tube with other modalities of treatment. The study did not include the chances of requirement of multiple modalities in the same patient. The database was limited to compare the effectiveness of T-tube in different age groups and patients with stenosis at different anatomical levels. These findings are mainly based on retrospective data.

## Conclusion

We encounter laryngotracheal stenosis in day-to-day practice, and as ENT surgeons, we should be competent to handle such challenging cases. Through this article, we conclude that silicon T-Tube for benign acquired laryngotracheal stenosis with Shiann Yann Lee’s technique is effective in most cases, safe with significantly less complication, and good acceptability and tolerance by the patient. The skills required to perform this surgery are easily achieved with a fast learning curve, with lesser chances of the requirement of an intensive care unit or high dependency unit. 

We want to conclude that silicon T-tube insertion is a safe and effective option even for those patients who are unwilling (in our case series due to financial issues) or unfit for major surgical intervention or other therapeutic options. However, further research and follow-up are needed amongst those who prefer to continue the T-tube of the long-term effectiveness of the same.
